# Assessing antibiotic effectiveness for reducing postoperative infectious complications in acute cholecystitis: a multicenter randomized controlled trial

**DOI:** 10.1097/JS9.0000000000002346

**Published:** 2025-03-28

**Authors:** Sung Eun Park, Tae Yoon Lee, Chang Ho Seo, Eui Soo Han, Tae Ho Hong

**Affiliations:** a Department of Hepato-biliary and Pancreas Surgery, Seoul St. Mary’s Hospital, College of Medicine, The Catholic University of Korea, Seoul, Republic of Korea; b Department of Surgery, Incheon St. Mary’s Hospital, College of Medicine, The Catholic University of Korea, Seoul, Republic of Korea; c Department of Surgery, Bucheon St. Mary’s Hospital, College of Medicine, The Catholic University of Korea, Seoul, Republic of Korea; d Department of Surgery, Uijeongbu ST. Mary’s Hospital, College of Medicine, The Catholic University of Korea, Seoul, Republic of Korea

**Keywords:** acute cholecystitis, anti-bacterial agents, cephalosporin, cholecystectomy, surgical wound infection

## Abstract

**Background:**

Patients with acute cholecystitis (AC) often receive antibiotics to reduce the risk of infectious complications after cholecystectomy. This study investigated the clinical significance of antibiotic use in patients with mild-to-moderate AC who required emergency laparoscopic cholecystectomy.

**Methods:**

This multicenter, double-blind, randomized controlled trial was conducted at four centers between February 2023 and January 2024. Patients with AC eligible for emergency laparoscopic cholecystectomy were randomly assigned to the antibiotic or placebo group. The antibiotic group received 1 g of intravenous cefazolin daily for three days during hospitalization and oral antibiotics for 4 days after discharge, whereas the placebo group received 10 mL of intravenous normal saline during their hospital stay. The primary endpoint was the rate of infectious complications.

**Results:**

An imputed per-protocol analysis of 370 patients (185 in each group) found comparable postoperative infection rates between the antibiotic group (7.6%, 14 patients) and placebo group (7%, 13 patients), showing no statistically significant difference (*P* = 0.842). Overall, the non-infectious complication rates did not differ significantly between the two groups: 21 (11.5%) cases in the antibiotic group vs. 30 (16.2%) cases in the placebo group (*P* = 0.591). Considering a non-inferiority margin of 10%, the absence of antibiotic treatment did not result in worse clinical outcomes than the antibiotic treatment.

**Conclusions:**

Administering antibiotics, even in sufficient doses, did not significantly reduce the risk of infectious complications in patients with AC compared to the group that did not receive antibiotics.

**HIGHLIGHTS**

- A multicenter, double-blind, randomized controlled trial was conducted on antibiotic effectiveness in acute cholecystitis.
- Patients with mild-to-moderate acute cholecystitis undergoing emergency laparoscopic cholecystectomy were randomly assigned to antibiotic or placebo groups.
- Postoperative infection rates were comparable between the antibiotic group (7.6%) and placebo group (7%).
- Considering a non-inferiority margin of 10%, absence of antibiotics did not result in worse outcomes.
- Intraoperative complications were the only significant risk factor for postoperative infectious complications.
- Administering antibiotics did not significantly reduce infectious complications in mild-to-moderate acute cholecystitis compared to no antibiotics.

## Introduction

Acute cholecystitis (AC) is a highly prevalent condition that frequently necessitates emergency laparoscopic cholecystectomy (LC)^[^1^]^. Patients with AC typically receive empirical antibiotic treatment perioperatively. Current guidelines recommend administering antibiotics for a period ranging from 24 hours before surgery to 7 days after the procedure^[^2^]^. AC commonly occurs because of obstruction of the cystic duct by gallstones, leading to cholestasis and inflammation of the gallbladder. In other words, as the initial trigger is a mechanical obstruction rather than a microbial source, early AC is not considered to be an infectious disease. If inflammation lasts longer than 72 hours after the onset of symptoms, there is a chance of developing a secondary bacterial infection. However, this only occurs in approximately 20% of acute calculous cholecystitis cases^[^3,4^]^. The use of antibiotics can lead to several negative outcomes. It is well known that extended courses of antibiotic treatment contribute to the development and spread of multidrug-resistant bacteria^[^5^]^. Moreover, extended periods of intravenous antibiotic administration are associated with increased hospitalization and healthcare costs^[^6^]^. The importance of selecting and administering empirical antibiotics before and after surgery to prevent infectious complications has been questioned. There are concerns about whether the choice of antibiotics and duration of their use can truly make a difference in reducing the risk of infection. Furthermore, it is widely believed that curbing the indiscriminate use of antibiotics can help minimize the side effects associated with their empirical use. According to randomized controlled trials conducted on the need for postoperative antibiotics in patients with mild or moderate AC who have undergone LC, the incidence of infectious complications after surgery ranges from 4% to 6.6% in the postoperative antibiotic group and 4% to 5.8% in the non-postoperative antibiotic group^[^1,5,7^]^. These studies found no significant differences in the rates of infectious complications between postoperative antibiotic and non-antibiotic groups. This suggests that postoperative antibiotics have no clinical effect on reducing postoperative infectious complications. These studies have indicated that administering empirical antibiotics once or twice during hospitalization might be insufficient to adequately treat a patient’s infectious condition, raising concerns regarding the effectiveness of this approach in preventing postoperative infectious complications. This multicenter, randomized controlled trial aimed to determine whether administering sufficient antibiotics could prevent postoperative infectious complications in patients undergoing emergency cholecystectomy for mild-to-moderate AC. We hypothesized that antibiotic administration would not significantly reduce the incidence of postoperative infectious complications, even when adequate antibiotics were administered during the treatment period, and that surgical intervention alone would be an effective treatment modality.

## Participants and methods

### Study participants and eligibility criteria

A multicenter, double-blind, controlled trial was conducted at four Catholic university hospitals (Seoul St. Mary’s Hospital, Incheon St. Mary’s Hospital, Bucheon St. Mary’s Hospital, and Uijeongbu St. Mary’s Hospital) in South Korea. The work has been reported in line with Consolidated Standards of Reporting Trials (CONSORT) Guidelines^[^8^]^. Patients admitted for surgery after AC were enrolled in this study from February 2023 to January 2024. The diagnosis of AC was based on the criteria described in the Tokyo Guidelines 2018^[^9^]^, with AC classified into three grades according to disease severity (Table 1). This study was approved by the Institutional Review Board of the hospital (XC22EIDI0041) and was registered at clinicaltrials.gov (trial number: NCT05339282). This study enrolled patients with AC grades I and II. However, grade II patients underwent additional classification. Patients with severe inflammation that had already dissolved the gallbladder (GB) boundaries, such as those with GB empyema, gangrenous cholecystitis, or GB perforation, were excluded. The authors proposed that inflammation extending beyond the destroyed GB boundary could be severe, potentially affecting nearby organs or tissues and leading to systemic manifestations even after removal of the primary GB lesion. Consequently, in cases of severe grade II AC, where inflammation has spread beyond the GB boundaries, appropriate antibiotic treatment might be justified. To address this issue, grade II AC was further categorized into grade IIa – AC with preserved GB wall structure – and grade IIb – GB wall structure destroyed, as observed on preoperative images, including cases of GB empyema, gangrenous cholecystitis, GB perforation, bile peritonitis, and pericholecystic abscess – as in a previous study^[^10^]^. This study focused exclusively on AC grades I and IIa cases. In patients considered high-risk due to clinically severe organ dysfunction or fatal comorbidities, drainage procedures were prioritized. These procedures included percutaneous transhepatic gallbladder drainage, followed by delayed cholecystectomy 4–6 weeks later. Patients who underwent these procedures were excluded from this study. Patients diagnosed with grade IIb AC during surgery, who showed different findings from preoperative imaging findings, were excluded from this study. The patients were started on postoperative antibiotic therapy. Prior to randomization, patients were excluded if they met any of the following criteria: immunodeficiency, concurrent operation on other organs, suspicion of malignancy, or a history of previous upper abdominal surgery. Additionally, patients were excluded if hollow organ injury was suspected, the common bile duct was explored, or the procedure was converted to laparotomy.

**Table 1:** Diagnostic criteria for acute cholecystitis according to the Tokyo Guidelines (Miura)

Severity grade	Criteria
Grade I	Does not meet the criteria of “severe” or “moderate” acute cholecystitis.
Mild	Can also be defined as acute cholecystitis in a healthy patient with no organ dysfunction and mild inflammatory changes in the gallbladder
Grade II	Associated with any one of the following conditions:
	Elevated white blood cell count (>18,000/mm^3^) Palpable tender mass in the right upper abdominal quadrant Duration of complaints >72 h Marked local inflammation (gangrenous cholecystitis, pericholecystic abscess, hepatic abscess, biliary peritonitis, emphysematous cholecystitis)
Moderate	
Grade III	Associated with dysfunction of any of the following organs/systems:
	Cardiovascular dysfunction (hypotension requiring treatment with dopamine > 5 μg/kg/minute or any dose of norepinephrine) Neurological dysfunction (decreased level of consciousness) Respiratory dysfunction (PaO_2_/FiO_2_ ratio < 300) Renal dysfunction (oliguria, creatinine > 2.0 mg/dL) Hepatic dysfunction (PT-INR > 1.5) Hematological dysfunction (platelet count < 100 000/mm^3^)
Severe	

PaO_2_/FiO_2_, Ratio of partial pressure of arterial oxygen to fraction of inspired oxygen; PT-INR, Prothrombin time-international normalized ratio.

### Study protocol and interventions

#### Surgical procedure

In this study, most LC procedures were performed using the three-trocar technique^[^11^]^. However, in certain special cases, a fourth trocar was inserted to facilitate the operation. All surgeries were performed by experienced surgeons who had completed more than 1000 cases of LC. Once the gallbladder was removed, it was placed in the specimen bag. The surgical site was thoroughly irrigated with saline until all gallbladder fragments and infected debris were completely eliminated. Subsequently, specimens were retrieved using an umbilical trocar. Routine drain placement was not performed, except in situations where there were concerns about potential bile leakage or bleeding. Following specimen retrieval, a culture study was conducted on samples taken from peripheral blood, gallbladder bile, and umbilical wounds to assess the microbiological profiles.

## Interventions

All patients who presented with AC at the emergency department were randomly assigned to either an antibiotic group (group A) or a placebo group (group B) before undergoing surgery. Once the diagnosis was confirmed, the research nurse administered the infusion according to the allocated group. Patients in group A received 1.0 g of 1st generation cephalosporin (cefazolin) as an empirical antibiotic, in accordance with the 2018 Tokyo guidelines^[^9^]^. In contrast, patients in Group B were administered 10 mL of intravenous normal saline in the emergency room. Cholecystectomy was typically performed within 24 h after randomization. After surgery, patients in group A continued to receive intravenous antibiotic (cefazolin) once daily as per the prescribed regimen, whereas those in group B received placebo injections of normal saline at the same frequency until their discharge from the hospital. Upon discharge, patients in group A were prescribed a third-generation cephalosporin (cefditoren, 100 mg three times daily) for an additional 4–5 days, completing a total antibiotic treatment course of 7 days. However, the patients in Group B did not receive any further treatment once they were discharged. Upon discharge, all patients were prescribed analgesics (NSAIDs) to manage pain. Hospital discharge was determined on the basis of the individual’s clinical condition. On the seventh day after discharge, all patients visited the outpatient department for general health checkup and wound status examination. Finally, to determine the presence of surgical site infection (SSI), the study investigator routinely conducted telephone interviews and assessed the presence of SSI 4 weeks after discharge.

### Criteria for protocol interruption in the placebo group

In group B, intravenous antibiotics were promptly initiated if any of the following infectious conditions or clinical alterations developed postoperatively. First, the patient exhibited persistent fever (>39°C) for >48 h after cholecystectomy without evidence of atelectasis. Second, newly emerging signs of peritonitis and hemodynamic instability were observed. Third, laboratory parameters, such as leukocytosis, worsened, as determined by a WBC count exceeding 18 000/mm^3^. Fourth, bacteremia was detected during hospitalization. Finally, any signs of clinical deterioration were noted within 48 h after surgery, based on the attending physicians’ judgment. These unusual cases were documented as protocol interruptions in Group B and were reclassified into a subgroup with postoperative infectious complications.

### Randomization

Informed consent was obtained from all participants at the time of hospital admission. A computer-generated table of random sampling numbers was created. An independent investigator, who was blinded to the study protocol, verified the randomized sampling number and assigned each patient to either the antibiotic (group A) or placebo (group B) group. Randomization was performed on-site at a 1:1 allocation ratio.

### Primary and secondary endpoints

The primary endpoint of this trial was the incidence of infectious morbidities, with a specific focus on surgical site infections (SSI) that occurred within 30 days of surgery. Table 2 provides the detailed definitions of these morbidities. In addition, this study evaluated several secondary endpoints, including the prevalence of bactibilia (presence of bacteria in the bile) and bacteremia (presence of bacteria in the blood). C-reactive protein (CRP) levels, leukocyte counts, non-infectious morbidities, length of hospital stay, and readmission rates were also assessed. Non-infectious morbidities encompassed a range of conditions, such as atelectasis, postoperative pancreatitis, choledocholithiasis, and prolonged diarrhea. Moreover, this study documented readmission cases that required radiologic intervention or surgical treatment following the initial surgery for any reason related to the cholecystectomy procedure. To maintain objectivity throughout this study, all parameters were prospectively gathered by independent investigators who were not directly involved in the current study.

**Table 2:** Definitions of infectious complications

Complication	Definition
Wound infection	
Superficial	Localized signs, such as redness, pain, heat, or swelling, at the site of the incision or by the drainage of pus
Deep	Presence of pus or an abscess, fever with tenderness of the wound, or separation of the edges of the incision exposing the deeper tissues
Organ or space infection	Fever and/or elevated CRP/WBC count and intra-abdominal fluid collection visualized by CT imaging or ultrasound
Pneumonia	Coughing or dyspnea, radiography with infiltrative abnormalities, or elevated infection parameters in combination with positive sputum culture
Urinary tract infection	Dysuria, elevated WBC count, and/or presence of nitrate in urine sediment in combination with a positive urine culture
Bacteremia	Presence of at least one positive hemoccult test result for the same pathogen

CRP, C-reactive protein; CT, Computed tomographic; WBC, White blood cell.

### Statistical analysis

To determine the sample size required for this study, a non-inferiority test was conducted. In a previous study^[^12^]^, the incidence of postoperative infectious complications in patients who underwent emergency LC was 15% in the antibiotic group and 17% in the placebo group. The non-inferiority margin was set as the upper limit of the confidence interval (CI) between the two groups, which was 11%. We determined this margin by using half of the postoperative infection rates reported in the literature. This approach aligns with FDA recommendations for anti-infective trials, which suggest a non-inferiority margin of about 10%. The clinical relevance of our chosen margin relates to the Altemeier classification, specifically for grade 2 (non-contaminated) cholecystectomies, where the expected postoperative infection rates without antibiotics typically range from 10% to 20%. Considering a dropout rate of 10%, 370 subjects (185 per group) were required. The criterion for establishing non-inferiority was that the upper limit of the two-sided 95% CI of the difference in the proportion of infections between the two groups should be lower than the non-inferiority margin. For patients not analyzed for the primary endpoint, including those who were lost to follow-up, an intention-to-treat analysis with multiple imputations was performed. Additionally, a per-protocol analysis was conducted, excluding patients in the placebo group who had missing data during the follow-up period. Continuous data were analyzed using the Student’s *t*-test. Categorical variables were compared using Fisher’s exact test or the χ^2^ test. Descriptive statistics were conducted using SPSS statistical package software (version 23.0; SPSS Inc., Chicago, IL, USA). Results are presented as the mean ± standard deviation. Univariate comparisons were performed using the log-rank test. Statistical significance was set at *P* < 0.05. All significant factors in the univariate analysis were entered into a multivariate logistic regression analysis to identify independent factors.

## Results

### Study enrollment and baseline characteristics

**Figure 1.**
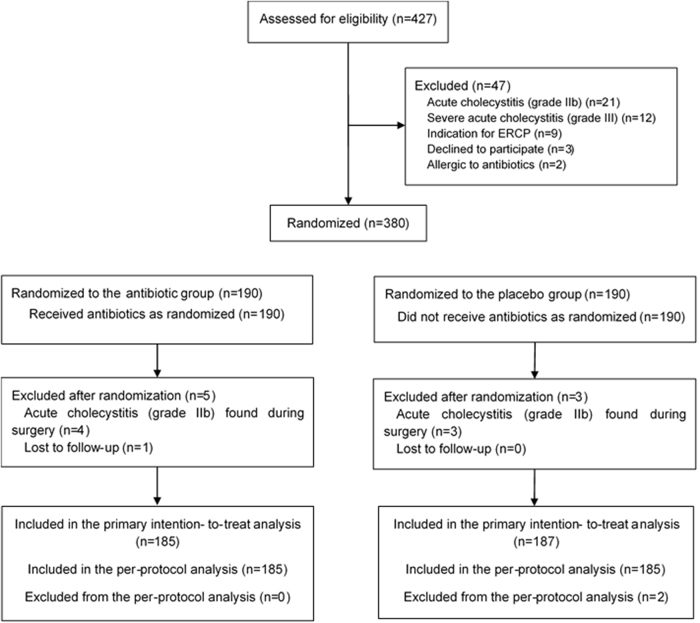
Schematic diagram of patient enrollment.

During the study period, 427 patients with AC visited the emergency department. Of these, 47 patients were excluded from this study for various reasons: 21 with AC grade IIb, 12 with severe AC (grade III), 9 with cholangitis, 3 who refused to participate, and 2 who were allergic to cefazolin. After randomization, 380 patients (190 in each group) were included in the intention-to-treat analysis. However, 10 patients were later excluded because they were found to have AC grade IIb during the operation, had bacteremia, or were lost to follow-up. Finally, 370 patients were included in the per-protocol analysis (Fig. 1). The study included patients aged between 18 and 75 years (mean age, 52 years). Among the participants, 110 (44.4%) were male and 137 (55.2%) were female. As shown in Table 3, the baseline demographic and clinical characteristics were comparable between the two treatment groups, indicating a well-balanced study design.

**Table 3:** Demographics and clinical characteristics of the study population

	Intention-to-Treat Analysis			Per-Protocol Analysis			
Characteristics	Antibiotics (*n* = 185)	Placebo (*n* = 187)	*P*	Antibiotics (*n* = 185)	Placebo (*n* = 185)	*P*
Age, yrs	51.2 ± 14.8	51.9 ± 14.16	0.517	51.2 ± 14.8	51.6 ± 14.02	0.779
Sex, *N* (%)			0.213			0.211
Male	91 (49.2)	79 (42.2)		91 (49.2)	79 (42.7))	
Female	94 (50.8)	108 (57.8)		94 (50.8)	106 (57.3)	
BMI	25 ± 4	24.9 ± 3.89	0.501	25 ± 4	25 ± 3.88	0.916
ASA score, *N* (%)			0.753			0.753
1	51 (27.6)	57 (30.5)		51 (27.6)	57 (30.8)	
2	121 (65.4)	119 (63.6)		121 (65.4)	117 (63.2)	
3	13 (7)	11 (5.9)		13 (7)	11 (5.9)	
CCI	1.5 ± 1.64	1.4 ± 1.5	0.532	1.5 ± 1.64	1.4 ± 1.49	0.406
Previous history of operation, *N* (%)	46 (25)	51 (27.3)	0.707	46 (25)	51 (27.6)	0.575
AC grade^a^ (%)			0.407			0.533
Grade I	95 (51.4)	89 (47.6)		95 (51.4)	89 (48.1)	
Grade IIa	90 (48.6)	98 (52.4)		90 (48.6)	96 (51.9)	
Preoperative laboratory values						
Leukocytes (/μL)	8885.1 ± 3090.09	8537.2 ± 1960.09	0.244	8885.1 ± 3090.09	8526.3 ± 1951.76	0.207
Segmented neutrophil (%)	66.1 ± 13.43	63.4 ± 10.03	0.064	66.1 ± 13.43	63.4 ± 10.05	0.051
C-reactive protein (mg/dL)	0.8 ± 1.12	0.6 ± 0.73	0.296	0.8 ± 1.12	0.6 ± 0.73	0.262
AST (U/L)	31 ± 24.94	27.9 ± 25.76	0.166	31 ± 24.94	27.9 ± 25.93	0.244
ALT (U/L)	36.1 ± 35.45	29.5 ± 29.6	0.153	36.1 ± 35.45	29.5 ± .29.75	0.058
Total bilirubin (mg/dL)	0.6 ± 0.32	0.6 ± 0.27	0.218	0.6 ± 0.32	0.6 ± 0.27	0.261
Direct bilirubin (mg/dL)	0.3 ± 0.13	0.3 ± 1.37	0.482	0.3 ± 0.13	0.2 ± 0.11	0.19

^a^ AC grade was defined according to the TG13 severity grading for AC. Grade I indicates mild AC which does not meet the criteria for grades II or III. Grade II is defined as moderate AC that is associated with any of the following features: elevated white blood cell count (>18,000/ mm3), presence of a palpable tender mass in the right upper abdominal quadrant, duration of complaints over 72 h, or marked signs of local inflammation such as gangrenous cholecystitis, pericholecystic abscess, hepatic abscess, biliary peritonitis, or emphysematous cholecystitis. We subdivided grade II AC into grade IIa (cases which presented with preserved GB wall structure, such as AC, gangrenous cholecystitis, or GB empyema) and grade IIb (cases in which the boundary of the GB was already destroyed before the operation, such as cases of GB perforation, bile peritonitis, or pericholecystic abscess). Grade III is the most severe form of AC and is associated with dysfunction of organs or systems. In this study, we excluded cases of AC grade IIb or II

BMI, body mass index; ASA, American Society of Anaesthesiologists; CCI, Charlson comorbidity index; AST, aspartate transaminase; ALT, alanine transferase; GB, gall bladder; AC, acute cholecystitis.

### Perioperative outcomes

The mean operation time, estimated blood loss, type of surgical procedure, and incidence of intraoperative complications showed no statistically significant differences between the two groups. Similarly, there was no significant difference in postoperative morbidity between the two groups (17.3% vs. 22.2%, *P* = 0.24). The incidence of non-infectious complications did not differ significantly between the two groups (11.5% vs. 16.2%, *P* = 0.591). Table 4 presents the perioperative outcomes of the two groups.

**Table 4:** Perioperative Outcomes of the study population

	Intention-to-Treat Analysis			Per-Protocol Analysis			
Variable	Antibiotics (*n* = 185)	Placebo (*n* = 187)	*P*	Antibiotics (*n* = 185)	Placebo (*n* = 185)	*P*
Operation time, min	37.9 ± 15.22	38.8 ± 15.21	0.66	37.9 ± 15.22	38.5 ± 14.9	0.693
EBL, cc	18.7 ± 17.2	15.5 ± 17.84	0.088	18.7 ± 17.2	15.6 ± 17.92	0.09
Operative procedure, *N* (%)			0.623			0.623
3-port LC	182 (98.4)	186 (99.6)		182 (98.4)	184 (99.5)	
4-port LC	3 (1.6)	1 (0.5)		3 (1.6)	1 (0.5)	
Intraoperative complication, *N* (%)			0.766			0.76
Bile spillage	6 (3.2)	6 (3.2)		6 (3.2)	5 (2.7)	
Other organ injury	0	0		0	0	
LOS, days	2.1 ± 0.47	2 ± 0.4	0.121	2.1 ± 0.47	2 ± 0.4	0.121
Postoperative morbidities, *N* (%)	32 (17.3)	42 (22.5)	0.212	32 (17.3)	41 (22.2)	0.24
Non-infectious complications, N (%)	21 (11.5)	30 (16)	0.188	21 (11.5)	30 (16.2)	0.591
Atelectasis	1 (0.5)	9 (4.8)		1 (0.5)	9 (4.9)	
diarrhea	9 (4.9)	15 (8)		9 (4.9)	14 (7.6)	
A. fib	0	2 (1.1)		0	2 (1.1)	
CVA	1 (0.5)	0)		1 (0.5)	0	
Ileus	0	2 (1.1)		0	2 (1.1)	
PONV	10 (5.4)	2 (1.1)		10 (5.4)	2 (1.1)	
Reoperation, *N* (%)	0	0	1.000	0	0	1.000
Re-admission, *N* (%)	0	1 (0.5)	1.000	0	1 (0.5)	1.000

EBL, estimated blood loss; LC, laparoscopic cholecystectomy; LOS, length of hospital stays; CVA, cerebrovascular accident.

### Postoperative infectious complications

In the imputed intention-to-treat analysis, postoperative infection rate was 7.6% (14 of 185) in group A and 7% (13 of 187) in group B, with an absolute difference of 0.98% (95% CI, 0.14% to 1.02%; *P* = 1.000). The per-protocol analysis yielded similar results, showing a postoperative infection rate of 7.6 % (14 of 185) in group A and 7 % (13 of 185) in group B, with an absolute difference of 0.98% (95% CI, 0.14% to 6.91%; *P* = 1.000). The non-inferiority margin was set to 11%. These results demonstrated that non-antibiotic treatment was not inferior to the administration of empirical antibiotics in preventing postoperative infectious complications. SSI, particularly superficial SSI, was the most common infectious complication, with no significant difference in SSI incidence between the two groups (7.6% in group A vs. 7 % in group B, *P* = 0.819). In group A, among 14 patients with SSI, 10 (5.4%) had superficial incisional SSI, 3 (1.6%) had deep incisional SSI, and 1 (0.5%) had an organ or space SSI (intra-abdominal abscess). Similarly, in group B, out of 13 patients with SSI, 10 (5.4%) had superficial incisional SSI, 2 (1.1%) had deep incisional SSI, and 1 (0.5%) had an organ or space SSI (intra-abdominal abscess). All patients with organ or space infections required radiological catheter drainage for intra-abdominal fluid collection. Table 5 summarizes the prevalence of infectious complications in both groups.

**Table 5:** Postoperative infectious complications of the study population and outcome of the cultures for patients from whom wound, bile, and blood samples were taken

	Intention-to-Treat Analysis			Per-Protocol Analysis			
Type of event	Antibiotics (*n* = 185)	Placebo (*n* = 187)	*P*	Antibiotics (*n* = 185)	Placebo (*n* = 185)	*P*
Infectious complications, *N* (%)	14 (7.6)	13 (7)	0.819	14 (7.6)	13 (7)	0.842
Surgical site infection, N (%)	14 (7.6)	13 (7)	0.767	14 (7.6)	13 (7)	0.787
Superficial incisional SSI	10 (5.4)	10 (5.3)		10 (5.4)	10 (5.3)	
Deep incisional SSI	3 (1.6)	2 (1.1)		3 (1.6)	2 (1.1)	
Organ/space SSI	1 (0.5)	1 (0.5)		1 (0.5)	1 (0.5)	
Distant infection, *N* (%)	0	0	1.000	0	0	1.000
Positive in culture, *N* (%)						
Wound	10 (5.4)	10 (5.3)	1.000	10 (5.4)	10 (5.4)	0.166
Bile	25 (13.5)	18 (9.6)	0.199	25 (13.5)	18 (9.7)	0.256
Blood	2 (1.1)	0	0.499	2 (1.1)	0	0.499

### Microbiological profile

The study protocol included the collection of wound, bile, and blood samples from all enrolled patients. Analysis of these samples revealed that 20 (5.4%) patients had a positive wound culture and 44 (11.6%) patients had a positive bile culture. Positive blood cultures were found in only two (1.1%) patients, both of whom were in Group A. No significant differences were observed in positive culture rates for wounds (*P* = 0.166), bile (*P* = 0.256), or blood (*P* = 0.499) between the two groups. The overall positive blood culture rate was 0.5% (Table 5). The most frequently isolated bacteria from wound cultures were *Staphylococcus* spp. (nine positive samples, 45%), with *Staphylococcus epidermidis* being the most common species within this family (six positive samples, 30%). In positive bile cultures, *Escherichia coli* was the most commonly identified bacterium (13 positive samples, 29.5%), followed by gram-positive bacilli, specifically *Enterococcu*s spp. (seven positive samples, 15.9%), and multiple microorganisms (six positive samples, 13.6%). Gram-negative bacteria were cultured from the blood samples of two patients, with one sample growing *Escherichia coli* and the other growing *Klebsiella pneumoniae* (Supporting Information. http://links.lww.com/JS9/E25).

### Predictive value of postoperative infectious complications

Univariate and multivariate analyses were performed (Table 6) to identify risk factors associated with postoperative infectious complications. Univariate analysis included 16 factors, of which three were found to be associated with the development of infectious complications: age (≥65 years), the presence of intraoperative complications (bile leakage or other organ injury), and whole operation time (WOT). Multivariate analysis identified intraoperative complications as independent risk factors for the development of postoperative infectious complications. Patients who experienced intraoperative complications, such as bile leakage or injury to other organs had a 21.79 times higher risk of developing infectious complications than those without intraoperative complications (95% CI: 1.76–269.32, *P* < 0.001).

**Table 6:** Univariate and multivariate analysis of risk factors for postoperative infectious complications

	Univariate analysis			Multivariate analysis		
	Odds ratio	95% CI	*P*	Odds ratio	95% CI	*P*
Age (≥65 yrs)	5.92	1.39–25.26	0.017	1.83	0.14–23.79	0.646
Gender (male)	0.68	0.17–2.82	0.728			
BMI (≥25 kg/m^2^)	0.76	0.18–3.24	1.000			
ASA score						
1	Reference					
2	1.26	0.13–12.29	1.000			
3	8.3	0.72–96.15	0.815			
Previous history of operation (yes vs. no)	2.59	0.64–10.54	0.23			
Non-antibiotic treatment						
Intention-to-treat	0.98	0.14–1.02	1.000			
Per-protocol	0.98	0.14–6.91	1.000			
AC Grade (IIa)	0.92	0.23–3.74	1.000			
Preoperative laboratory values						
Leukocytes (/μL)	1.02	0.18–5.76	0.228			
Segmented neutrophil (%)	0.97	0.89–1.07	0.211			
C-reactive protein (mg/dL)	1.5	0.72–3.1	0.076			
Intraoperative complication (yes vs. no)	1.08	0.14–8.63	<0.001	21.79	1.76–269.32	<0.001
Operation time, min	0.99	0.97–1.01	0.001	1.02	0.97–1.08	0.401
EBL, cc	0.92	0.81–1.05	0.837			
Positive blood culture	0.99	0.98–1.00	1.000			
Positive bile culture	8.08	1.12–58.91	0.066			
Positive wound culture	6.39	0.63–64.51	0.192			

BMI, body mass index; ASA, American Society of Anesthesiologists; AC, acute cholecystitis; EBL, estimated blood loss; CI, confidence interval.

## Discussion

Our multicenter, double-blind, randomized controlled trial demonstrated that full-dose administration of empirical antibiotics did not significantly reduce the incidence of postoperative infectious or non-infectious complications in patients undergoing early emergency LC for mild-to-moderate (grade I and IIa) AC. No statistically significant differences were observed in the hospital admission or readmission rates between the intervention and control groups. Multivariate analysis identified intraoperative complications as the only significant risk factor of postoperative infectious complications in patients with AC. The postoperative clinical course was not significantly influenced by either the assigned treatment protocol or the presence of pathogenic organisms in cultures. Antibiotics used to treat AC primarily target enteric gram-negative bacteria found in bile, such as *Escherichia coli, Klebsiella*, and *Enterobacter*, as well as Gram-positive bacteria, such as *Enterococcus*. These antibiotics also play an adjunctive role in reducing the risk of postoperative infections^[^3^]^. However, our study found that the most common postoperative infectious complication was superficial SSI, with wound cultures predominantly showing normal skin flora, rather than enteric pathogens. This finding suggests a lack of evidence regarding the conventional use of antibiotics in patients with AC. Our study focused on patients with grade I or IIa AC, characterized by localized inflammation and well-preserved gallbladder walls. At this stage, AC is primarily caused by mechanical obstruction of the gallbladder neck and the cystic duct by gallstones. If bile stasis persists for >72 hours after symptom onset, it can progress from inflammation to infection. In such cases, inflammatory AC can develop into complicated infectious stages, such as gallbladder perforation, biliary peritonitis, or sepsis, for which antibiotic treatment may be necessary^[^13-15^]^. However, we minimized the risk of infectious complications by performing early cholecystectomy within 12–24 hours after diagnosis. Our study revealed that the use of full-dose antibiotics offers no significant advantage in preventing postoperative infectious complications. More importantly, we found that surgical management alone was sufficient to treat patients with AC (grade I and IIa). AC severity was classified according to several established guidelines. This stratification is fundamental for clinical practice, as it informs prognostic assessments and guides therapeutic decision-making. In our analysis of 406 patients with AC who visited the emergency department, excluding those who required percutaneous transhepatic gallbladder drainage (PTGBD) rather than surgical treatment, the distribution of severity grades was as follows: grade I, 184 (45.3%) patients; grade IIa, 189 (46.6%) patients; and grade IIb, 33 (8.1%) patients. Our findings showed that most patients who required emergency cholecystectomy presented with grade I or IIa AC. In a study by Yokoe, *et al*, 34.1%, 55.6%, and 15.3% of patients with AC were categorized as grades I, II, and III, respectively. Among the grade II cases, further histopathological analysis showed that 65.8% were classified as grade IIa, while 34.2% were grade IIb. Notably, when grade III patients who typically required PTGBD prior to surgical intervention were excluded, the majority (79.6%) of AC patients requiring surgery were found to be grade I and IIa patients^[^16^]^. These findings suggest that most patients presenting to the emergency room with AC are in an inflammatory rather than infectious state, indicating that surgical treatment alone may be sufficient for effective management. Furthermore, given that the treatment needs are different, it may be beneficial to introduce more detailed subdivisions within Grade II of the Tokyo guidelines. Current clinical guidelines advocate a limited course of antibiotic therapy, not exceeding 4 days in duration, for patients with AC who undergo cholecystostomy^[^2^]^. Previous studies have investigated the efficacy of preoperative empirical antibiotics in preventing postoperative infectious complications in patients undergoing emergency LC for mild-to-moderate AC^[^10,12^]^. These studies suggest that the absence of preoperative antibiotic treatment did not significantly affect the incidence of postoperative infectious complications. However, a brief duration of antibiotic administration, typically once or twice before surgery, may be insufficient to effectively treat the infection. Our study aimed to address these limitations and provide definitive evidence on the role of antibiotics in managing AC. By implementing a more comprehensive approach to antibiotic administration and focusing on specific AC grades, we sought to elucidate the true impact of antibiotic therapy on postoperative outcomes. We excluded patients with AC grade IIb and III from our study, so our findings should not be applied to these patients because we lacked sufficient evidence. Therefore, further studies are needed to assess the impact of antibiotic use in these populations. Although our study had compelling findings, it had some limitations. First, the rate and severity of complications were relatively low, with only two cases of sepsis. This likely reflects the characteristics of the medical environment in Korea, where the time from admission to cholecystectomy is relatively short, and surgical delays are rare. As a result, the rapid removal of the infection source might have contributed to the low incidence of severe systemic infections. Second, the classification of AC between grades IIa and IIb might have varied among the participating centers, potentially affecting patient selection. To minimize this subjectivity, we provided standardized visual criteria for grade assessment. Finally, we did not perform an analysis based on host factors, such as comorbidities, advanced age, or immunocompromised status. Therefore, we could not provide targeted guidelines for antibiotic selection in these groups. We plan to conduct further studies to determine whether antibiotic selection before LC should be tailored to these host factors.

In conclusion, empiric antibiotic administration might be unnecessary in patients diagnosed with mild-to-moderate AC, specifically grades I and IIa, with preserved gallbladder wall integrity. This is particularly relevant, because timely surgical management can potentially eliminate the need for prophylactic antibiotics. Forgoing antibiotic treatment did not appear to increase the risk of postoperative infectious complications in these cases. This study found that intraoperative complications were the only factors that significantly contributed to the development of postoperative infectious complications in patients with AC. This study contributes to the ongoing debate regarding the use of antibiotics for AC. This information may inform future clinical practice guidelines.

## Data Availability

Research data supporting the findings of this study are available from the corresponding author upon reasonable request. These data are not publicly available because of privacy or ethical restrictions.

## Research registration unique identifying number (UIN)

Hospital Institutional Review Board: XC22EIDI0041. Clinicaltrials.gov Identifier: NCT05339282.
